# Suicide prevention project with young people in the Rocinha slum in Rio de Janeiro

**DOI:** 10.1192/j.eurpsy.2024.1622

**Published:** 2024-08-27

**Authors:** C. Jorgejaber, V. Soares, P. Zanelatto, A. Muniz, A. H. G. Hollanda

**Affiliations:** Pesquisa, Clinica Jorge Jaber, Rio de Janeiro, Brazil

## Abstract

**Introduction:**

The 2019 WHO report on suicide warned of a serious public health problem. It was found that suicide is a serious problem for global public health, causing approximately 703 thousand deaths every year. Self-extermination is among the leading causes of death worldwide, with more deaths than from malaria, HIV/AIDS, breast cancer, war and homicide. More than one in every 100 deaths (1.3%) in 2019 were the result of suicide.Suicide is the fourth leading cause of death in older adolescents (15–19 years). Risk factors are multifaceted and include harmful use of alcohol, which includes abuse during childhood, stigma against seeking help, barriers to accessing care and means of suicide.The total number of deaths due to self-extermination registered in the adolescent population in the period from 2016 to 2021 was 6,588. According to the WHO director-general, “attention to suicide prevention is even more important now, after many months of living with the pandemic and many of the risk factors, such as loss of employment, financial stress and social isolation, still very present.” Therefore, suicide prevention work with young adolescents in Rocinha, one of the largest slums in Rio de Janeiro, is extremely important, given the increase in suicide rates and mental health problems in this age group. This approach must be thoughtful, culturally sensitive, and involve a range of strategies to address the complex issues affecting adolescents in the community. The Community of Rocinha was chosen to host this prevention project.

**Objectives:**

Create a preventive event by surveying participants’ opinions, integrating, welcoming and deconstructing stigmas about suicide.

**Methods:**

This study investigated, in a population of 140 young adolescents with cultural differences in a theater class, their level of knowledge regarding relevant information about suicide. A structured questionnaire was presented and answered before and after a lecture, resulting in a class at the end, carried out by the young participants themselves. The scenes were filmed and a film produced. This dynamic process also included the distribution of a shirt alluding to the fact, making the participants multiplier references.

**Results:**

The results of the lecture showed a significant improvement in mental health awareness and willingness to seek help among young people, totaling a 20% increase in knowledge.

**Conclusions:**

Students attended the event in significant numbers, taking into account that the slum had a curfew due to armed conflict. The results of the lecture showed a significant improvement in mental health awareness and willingness to seek help among young people, totaling a 20% increase in knowledge. The young people reported a feeling of support and belonging to the community, highlighting the importance of the debate in a final lecture given by them.

**Disclosure of Interest:**

None Declared

